# Towards a new vision of PaNET: enhancing reasoning capabilities for better photon and neutron data discovery

**DOI:** 10.1107/S1600577525005272

**Published:** 2025-07-30

**Authors:** Terence Tan, Balázs Bagó, Sebastian Busch, Renaud Duyme, Guillaume Gaisné, Alejandra Noemí González Beltrán, Heike Görzig, Giannis Koumoutsos, Rolf Krahl, Paul Millar, Carlo Minotti, Melanie Nentwich, Lajos Schrettner, Kirsty Syder, Philippe Rocca-Serra, Susanna-Assunta Sansone, Stephen P. Collins

**Affiliations:** a Diamond Light Source Didcot OxfordshireOX11 0DE United Kingdom; b University of Oxford Oxford e-Research Centre (OeRC) Keble Road OxfordOX1 3QG United Kingdom; c ELI-HU Research and Development Non-Profit, Wolfgang Sandner utca 3, H-6728Szeged, Hungary; d German Engineering Materials Science Centre (GEMS) at Heinz Maier-Leibnitz Zentrum (MLZ), Helmholtz-Zentrum Geesthacht GmbH, Lichtenbergstrasse 1, 85747Garching bei München, Germany; e European Synchrotron Radiation Facility 38043Grenoble France; f Computing Division, UK Atomic Energy Authority, Abingdon, OxfordshireOX14 3DB, United Kingdom; g Helmholtz-Zentrum Berlin für Materialien und Energie Hahn-Meitner-Platz 1 14109Berlin Germany; h Deutsches Elektronen-Synchrotron DESY Notkestr. 85 22607Hamburg Germany; i Paul Scherrer Institute Forschungsstrasse 111 5232Villigen Switzerland; ESRF – The European Synchrotron, France

**Keywords:** PaNET ontology, experimental techniques, FAIR principles, ExPaNDS, PaNOSC, open science, data catalogues

## Abstract

The Photon and Neutron Experimental Techniques (PaNET) ontology was released in 2021 as an ontology for two major European research infrastructure communities. It provides a standardized taxonomy of experimental techniques employed across the photon and neutron scientific domain, and is part of a wider effort to apply the FAIR (findable, accessible, interoperable, reusable) principles within the community. Specifically, it is used to enhance the quality of metadata in photon and neutron data catalogue services. However, PaNET currently relies on a manual definition approach, which is time consuming and incomplete. A new structure of PaNET is proposed to address this by including logical frameworks that enable automatic reasoning as opposed to the manual approach in the original ontology, resulting in over a hundred new technique subclass relationships that are currently missing in PaNET. These new relationships, which are evaluated by the PaNET working group and other domain experts, will improve data catalogue searches by connecting users to more relevant datasets, thereby enhancing data discoverability. In addition, the results of this work serve as a validation mechanism for PaNET, as the very process of building the logical frameworks, as well as any incorrect inferences made by the reasoner, has exposed existing issues within the original ontology.

## Abstract 2

This evolution of PaNET builds on the initial foundational work, which established the infrastructure and incorporated domain expert knowledge, by introducing logical frameworks that provide enhanced reasoning capabilities, and is proposed to address the limitations of the current version of the ontology. This under-the-hood development has resulted in a more complete and robust knowledge representation system for use in data catalogue services within the photon and neutron community.

## 1. Introduction

The Photon and Neutron Experimental Techniques (PaNET) ontology (Collins *et al.*, 2021 ▸) provides a standardized taxonomy of experimental methods employed across the photon and neutron (PaN) research community. Developed as part of the Horizon 2020 European Open Science Cloud Photon and Neutron Data Service (ExPaNDS) project, in close collaboration with its sister Photon and Neutron Open Science Cloud (PaNOSC) initiative, PaNET was created alongside NeXusOntology (da Graça Ramos, 2021 ▸) and PaNmapping (Görzig, 2021 ▸) to support data catalogue services for European PaN national research infrastructures. This ontology represents progress towards implementing the FAIR (findable, accessible, interoperable, reusable) principles (Wilkinson *et al.*, 2016 ▸), which are a set of guidelines and best practices for data management to enhance the value of digital resources and facilitate reuse by both humans and machines, within the PaN scientific domain. The ultimate aim is to facilitate more effective data cataloguing, making PaN scientific data more discoverable in accordance with the principles.

### 1.1. Ontology, taxonomy and controlled vocabulary

PaNET was designed as an ontology, which means that it formally defines experimental techniques and their relationships. Within this ontology, the taxonomy refers specifically to the hierarchical classification (in the form of parent–child relationships, also known as direct subclass relationships) of the techniques, while the controlled vocabulary consists of the standardized set of unique PaN technique terms. The taxonomy provides a clear graph-like representation of subclass relationships that is amenable to visualization, while the controlled vocabulary ensures consistency and reduces ambiguity in how techniques are referenced. The ontology can be thought of as encompassing these two components while including additional ontological elements such as equivalence relationships. Users interact primarily with the comparatively simpler and more interpretable taxonomy and controlled vocabulary, while the broader ontological framework remains mostly hidden.

### 1.2. Role within PaN community

Data curators use the controlled vocabulary defined in PaNET to tag experimental sessions or datasets (a single experimental session can include multiple datasets and *vice versa*) with the appropriate PaNET terms, enriching metadata and providing a clearer context in accordance with the FAIR principles. The addition of these metadata also improves data discoverability, which aids in PaN data catalogue searches. While plain text searches of technique names may suffice in certain situations, formalizing these techniques in the form of an ontology enables more versatile search functionalities, such as alternate label matching. Most importantly, the subclass relationships between techniques, which are inherent to the taxonomic aspect of the ontology, make it possible to search for general terms but still get specific terms, *i.e.* they enable semantic searching.

For example, the technique class ‘multiwavelength anomalous diffraction’ in PaNET has the alternate label ‘MAD’, and is a subclass of ‘x-ray probe’, ‘macromolecular crystallography’, ‘single crystal diffraction’, and ‘atomic core excitation’. A dataset tagged with ‘multiwavelength anomalous diffraction’ could still be found by searches for any of the latter’s parent classes or its alternate label ‘MAD’. This significantly increases the number of avenues for the data to be discovered, thereby enhancing their discoverability. This is useful for users who may not know the exact term for the techniques they are searching for, and also allows users to come across relevant datasets they were not intentionally looking for.

The PaNOSC federated search service (Richter, 2022 ▸), available on the PaNOSC Data Portal, takes advantage of these enhanced metadata to augment its search functionality by utilizing the PaNET controlled vocabulary as parameters to search for relevant datasets.

PaNET greatly benefits from involvement of experts from photon and neutron facilities. These experts are encouraged to propose and discuss changes collaboratively with their peers to ensure the accuracy and validity of their contributions.

### 1.3. Notation conventions

In this paper, the different types of classes and object properties (classes are distinct from object properties) in PaNET are presented using different bracket notations to distinguish between them:

(i) Angle brackets <…> denote technique classes.

(ii) Square brackets […] denote classes that represent PaN-related concepts.

(iii) Parentheses (…) denote object properties.

(iv) Curly brackets {…} denote anonymous classes (Section 3.3).

The labels for technique classes have been revised in the new structure of PaNET. These labels are distinct from the Internationalized Resource Identifiers, which are unique identifiers. In contrast, the labels are typically human readable and not necessarily unique. To clarify that these classes represent techniques, the suffix ‘technique’ has been added to their labels. For example, <x-ray diffraction> has been changed to <x-ray diffraction technique>. For consistency, this paper uses the new labels even when referring to techniques in the context of the current structure of PaNET, as both versions of a label denote the same underlying class.

Additionally, diagrams use ellipses to represent classes and rectangles to represent object properties.

### 1.4. High-level overview

**Figure 1:**
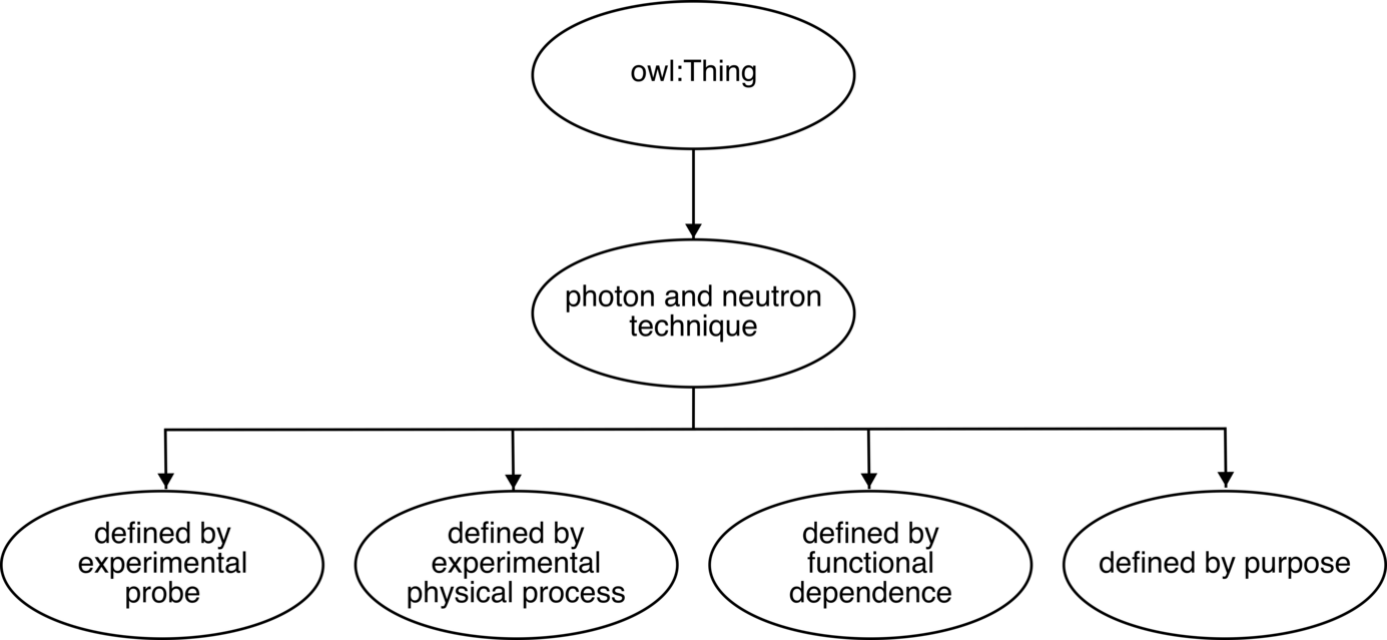
Current top-level view of PaNET. Each of these four technique classes represents a particular implied relationship, with subclasses not shown here. There are nearly 400 classes.

PaNET was created using the Web Ontology Language (OWL) (W3C OWL Working Group, 2012 ▸). As shown in Fig. 1 ▸, its structure comprises four top-level superclasses that serve as the ontology’s cornerstones: <defined by experimental probe>, <defined by experimental physical process>, <defined by functional dependence> and <defined by purpose>. Each of these superclasses groups technique classes based on a relationship to a distinct concept. For example, <defined by experimental probe> represents the set of techniques that can be defined by the type of probes that are involved. These relationships, which were chosen to be conceptually independent from one another to minimize overlap, are implied only by name and not made explicit in PaNET. In total, the ontology encompasses nearly 400 classes representing various techniques.

### 1.5. Limitations

PaNET was structured entirely through manually defined subclass relationships by domain experts. Each of these relationships was individually identified and explicitly defined based on their expert knowledge of PaN concepts. This manual classification approach resulted in a comprehensive taxonomy, but in the first instance lacked the formal logical structures that reasoning engines such as *HermiT* (Glimm *et al.*, 2014 ▸) can take advantage of to produce automated inferences. Reasoning and inferring in this context refer to the ability of a system to infer new knowledge from explicitly stated facts using formal logic. This is distinct from query languages such as SPARQL (SPARQL Working Group, 2013 ▸), which are used to retrieve existing information from an ontology.

For example, considering the well known pizza ontology, a manual approach might classify both ‘Pizza’ and ‘Pepperoni Pizza’ as subclasses of ‘Food’ based on human judgment but overlook the fact that ‘Pepperoni Pizza’ is also a subclass of ‘Pizza’. In contrast, a reasoner, given the formal definitions of these classes, could infer not only the same relationships as the manual approach but also additional ones, including the missing relationship between ‘Pepperoni Pizza’ and ‘Pizza’, by identifying hierarchical connections that were not explicitly stated. Consequently, PaNET is currently dependent on the completeness and accuracy of the human-specified relationships. Indeed, use of the ontology since its release has revealed missing subclass relationships, such as <x-ray diffraction technique> and <x-ray scattering technique>, highlighting the presence of gaps within PaNET.

Additionally, all relationships between classes in the original ontology are represented solely through these direct subclass axioms. This approach left important conceptual connections between techniques and their defining characteristics, such as the probes used, implicit in the class-naming conventions of many of the techniques rather than being formally represented in the ontology, as noted by the original architects of PaNET (Collins *et al.*, 2021 ▸). For example, <photon probe technique> and <x-ray probe technique> each imply a relationship to photon probes and X-ray probes, respectively. However, the probe classes and the relationships are not formally represented in PaNET. This limitation affects the ontology’s semantic richness.

Both the missing subclass relationships and the reduced semantic richness undermine the findability of technique terms and, by extension, datasets tagged with these terms. For example, searching for <x-ray scattering technique> and all its subclasses will fail to return <x-ray diffraction technique>. Similarly, it would not be possible to find the latter technique class by searching for techniques that use X-ray probes, since the relationship to X-ray probes is not explicitly represented currently. This ultimately hinders the performance of PaN data catalogue services or any other system that relies on PaNET.

## 2. Motivation

As stated earlier, the current structure of PaNET relies completely on the manual definition of subclass relationships, which introduces several challenges: it is time consuming, incomplete and lacks semantic richness. This work addresses these limitations by making the implied relationships within PaNET explicit, and leveraging them along with stronger logical statements (Section 3.4) as building blocks for a more robust and extensible ontology. As a result, this enables reasoners to perform automated inferences, eliminating the need for the manual approach. Moreover, the reasoner is able to infer subclass relationships that are overlooked in the current ontology. Using the same example from before, the reasoner is able to infer that <x-ray diffraction technique> is a subclass of <x-ray scattering technique> in this new structure of PaNET. The inference process is fully explained in Appendix *A*[App appa]. In most cases, the correctness (or otherwise) of these new subclass relationships is obvious and can be dealt with by the PaNET group. For anything more subtle, the group has access to domain experts at numerous facilities. This approach not only improves the completeness of the ontology but also increases its utility for the PaN community by creating a more robust knowledge representation system.

Crucially, these improvements to the underlying architecture of the ontology do not change what is being shown to users; they will only be exposed to the technique subclass relationships as before, albeit with about a hundred additional inferred relationships. This ensures that PaNET remains accessible to users who may be unfamiliar with OWL ontologies, while benefitting from a more comprehensive and inferentially powerful underlying structure. As a result, data catalogue services will enjoy improved performance, enhancing the discoverability of datasets while making it easier for researchers to find the data they need.

Furthermore, this work serves as a validation mechanism for PaNET, as the very process of building the logical frameworks to enable enhanced reasoning capabilities, as well as any incorrect inferences made by the reasoner, has exposed existing issues within the ontology.

## 3. Methodology

**Figure 2:**
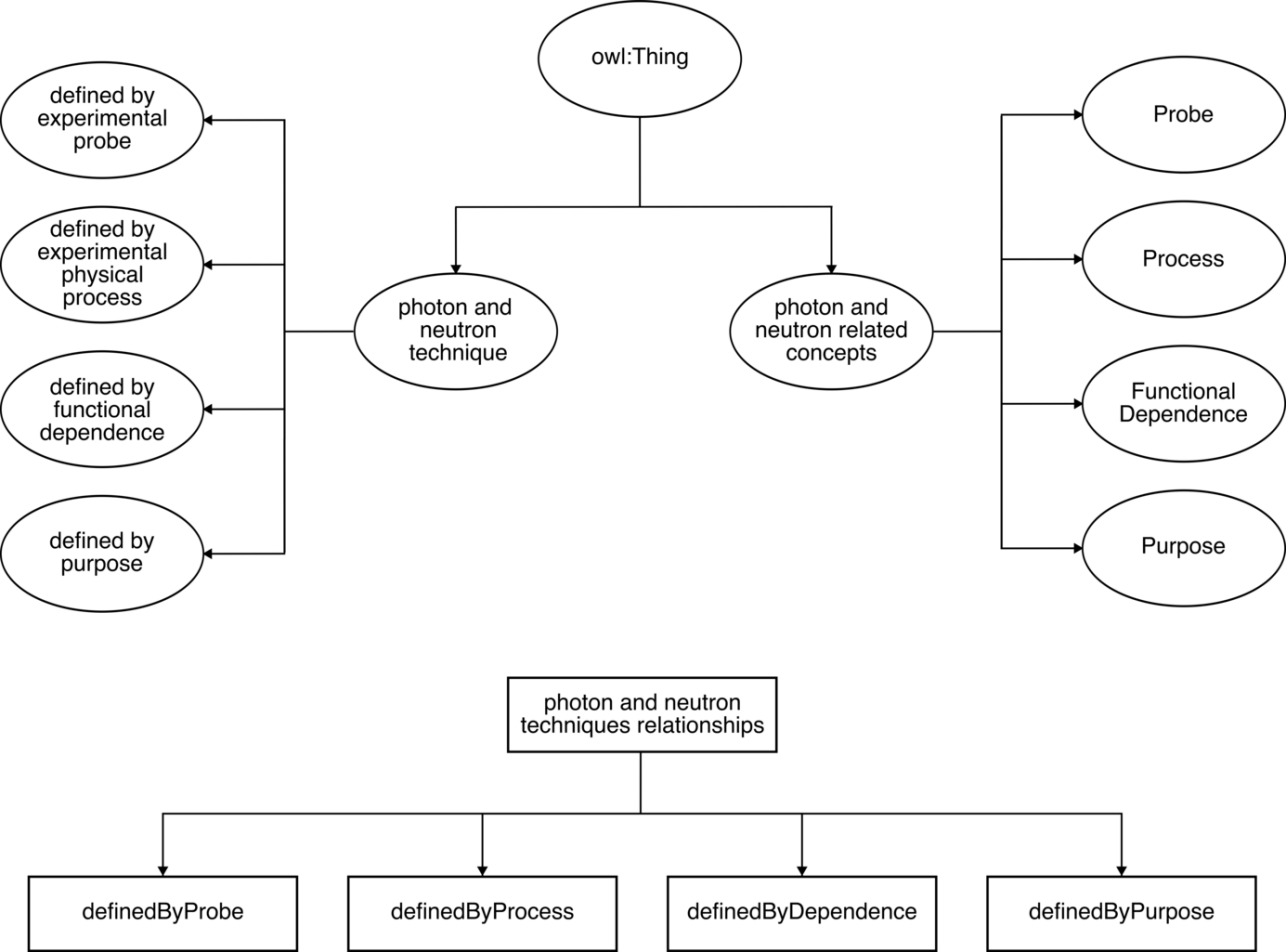
Top-level view of the new structure of PaNET. Four object properties and the associated PaN-related concept classes have been added. Similar to the technique classes, each of the four concept superclasses represents a broader hierarchy, with over a hundred additional subclasses in total not shown here. The ellipses represent classes while the rectangles represent object properties; the two are distinct types of ontological elements.

In order to build the logical structure needed for automated inferencing, four object properties and the corresponding relevant classes have been added to the new structure of the ontology, as shown in Fig. 2 ▸. The four object properties are (definedByProbe), (definedByProcess), (definedByDependence) and (definedByPurpose), and they characterize the relationships currently implied in PaNET. The new classes represent the PaN-related concepts (Section 3.1) to which the object properties (Section 3.2) map from the technique classes that exist in the ontology currently. Although Fig. 2 ▸ only shows four concept classes, each of these encompasses a range of more specific subclasses that are not depicted for simplicity. Including [photon and neutron related concepts], there are a total of 128 concept classes.

Existential property restrictions, also known as existential quantifications in the context of OWL (Hitzler *et al.*, 2012 ▸), are used to define anonymous classes (Section 3.3). An existential property restriction specifies that there exists at least one relationship (via a specific property) to a certain class. Anonymous classes are any classes that are not explicitly named.

Some technique classes are then defined as equivalent to these anonymous classes, *e.g.* <refraction technique> has been made equivalent to {(definedByProcess) **some** [refraction]}. The keyword **some** denotes existential restrictions; see Section 3.3. Some other technique classes have been made equivalent to the intersection of their parent technique classes, representing stronger logical statements (Section 3.4) by replacing the original subclass axioms with equivalence axioms,*e.g.* <x-ray refraction imaging technique> has been made equivalent to the intersection of <refraction technique>, <x-ray probe technique> and <imaging technique>. The remaining technique classes have simply been kept the same, *e.g.* <natural linear dichroism technique> being a subclass of <linear dichroism technique>. These changes enable the reasoner to automatically derive the subclass relationships that were manually defined in the current structure of PaNET, as well as subclass relationships that are missing from the ontology.

All changes have been implemented in the *ROBOT* (Jackson *et al.*, 2019 ▸) spreadsheet representation of PaNET, rather than in *Protégé* (Musen & Protégé Team, 2015 ▸). *ROBOT* is primarily a command-line tool for automating ontology development tasks; it provides a wide range of functionalities including ontology editing, conversion and validation. In contrast, *Protégé* is a graphical user interface-based OWL ontology development environment. *ROBOT* has been chosen as it is a more readable and interpretable format compared with the interface of *Protégé*. In addition, the PaNET source file is in CSV format, making the spreadsheet a more suitable working environment for these modifications. The updated spreadsheet was then exported as a CSV file and subsequently converted to an OWL file using the template command of the *ROBOT* tool.

### 3.1. PaN-related concept classes

The [photon and neutron related concepts] class has been added to the new structure as the superclass of all PaN-related concept classes. Since many technique classes imply their underlying concepts through their respective labels, systematic analysis of each technique class had to be carried out to identify which technique has an associated concept, and what those concepts are. This analysis revealed that the technique classes can be split into two groups:

(i) Core techniques. Each of these techniques implies a fundamental concept that should be explicitly represented as a concept class. For example, <refraction technique>, which denotes the group of techniques that involve the process of refraction, has a counterpart concept class [refraction], which refers to the refraction process itself.

(ii) Non-core techniques. These techniques do not have direct counterpart concept classes. An example would be <x-ray refraction imaging technique>.

In total, the new structure of PaNET contains 128 concept classes, including the [photon and neutron related concepts] class. These concept classes inherit the hierarchical structure of the technique classes from which they are derived. Using the earlier example of refraction, the [refraction] class is a child class of [Process] in the proposed structure of PaNET since the <refraction technique> class is currently a child class of <defined by experimental physical process> in PaNET.

### 3.2. Object properties

Four object properties have been added: (definedByProbe), (definedByProcess), (definedByDependence) and (definedByPurpose). Their domains have been set to <photon and neutron technique>, and their ranges are [Probe], [Process], [Functional Dependence] and [Purpose], respectively. In other words, they map a technique class to a PaN-related concept class.

In OWL ontology design, object properties can be assigned specific characteristics such as functional, inverse functional, transitive, symmetric, asymmetric, reflexive and irreflexive. For the four object properties introduced above, only the asymmetric and irreflexive characteristics have been applied, as the relationships represented by these properties do not inherently exhibit the other characteristics. A brief definition of each characteristic, adapted from OWL documentation (Hitzler *et al.*, 2012 ▸), is provided below, along with a justification for its inclusion or exclusion.

(i) Functional. If an object property is functional, then for any given individual *A*, there can only be at most one distinct individual *B* such that *A* is related to *B* through that property. For example, ‘hasUniqueID’ would be a functional property since each individual can only have at most one unique ID. None of the properties have been asserted to be functional since a technique may be associated with multiple PaN-related concepts.

(ii) Inverse functional. If a property is inverse functional, then its inverse property is asserted to be functional. An inverse property is essentially the flipped version of the original property. For example, the ‘hasUniqueID’ property would have the inverse property ‘isIdentifiedBy’, which is functional since each ID can only be linked back to at most one person. This makes ‘hasUniqueID’ inverse functional. ‘isIdentifiedBy’ also happens to be inverse functional because ‘hasUniqueID’ is functional as well. Inverse properties exist regardless of whether they have been explicitly declared. The inverse properties of the four object properties are not functional because a PaN-related concept can be associated with multiple techniques.

(iii) Transitive. Given a particular transitive relationship, if individual *A* is related to individual *B* and *B* is related to an individual *C*, then *A* is related to *C*. One example would be ‘hasAncestor’. As mentioned earlier, the PaNET object properties have been asserted to only map from technique classes to concept classes, and these two types of classes are expected to be disjoint. Hence, the properties cannot be transitive.

(iv) Symmetric/asymmetric. A symmetric property has itself as its inverse property. An example of a symmetric property would be ‘hasPartner’. Again, the four PaNET properties map from techniques to concepts. Thus, they cannot be symmetric and have been asserted to be asymmetric.

(v) Reflexive/irreflexive. If a property is reflexive, every individual in its domain must be related to itself through that property. Similar to before, the four object properties relate technique classes to concept classes and therefore cannot be reflexive. As a result, the properties have been set to be irreflexive.

### 3.3. Anonymous classes

Anonymous classes refer to any unnamed classes in OWL. They can be property restrictions, *e.g.* the unions of classes, or the intersections of classes (Bechhofer *et al.*, 2004 ▸). These classes are not explicitly named in an ontology but exist in its axioms.

In the current structure of PaNET, these classes manifest mainly in the form of intersections; when a class is asserted to be a subclass of multiple parent classes, the intersection of those parent classes forms an anonymous class.

In the proposed new structure of PaNET, existential property restrictions are used to enforce necessary relationships between the core technique classes and their respective concept classes. Each restriction defines an anonymous class consisting of individuals that are related to at least one instance of the concept class via the object property. For example, the anonymous class {(definedByProcess) **some** [refraction]} represents a class that is defined by at least one process of the type refraction. In both *ROBOT* and *Protégé*, the keyword **some** denotes existential restrictions.

### 3.4. Stronger logical statements

**Figure 3:**
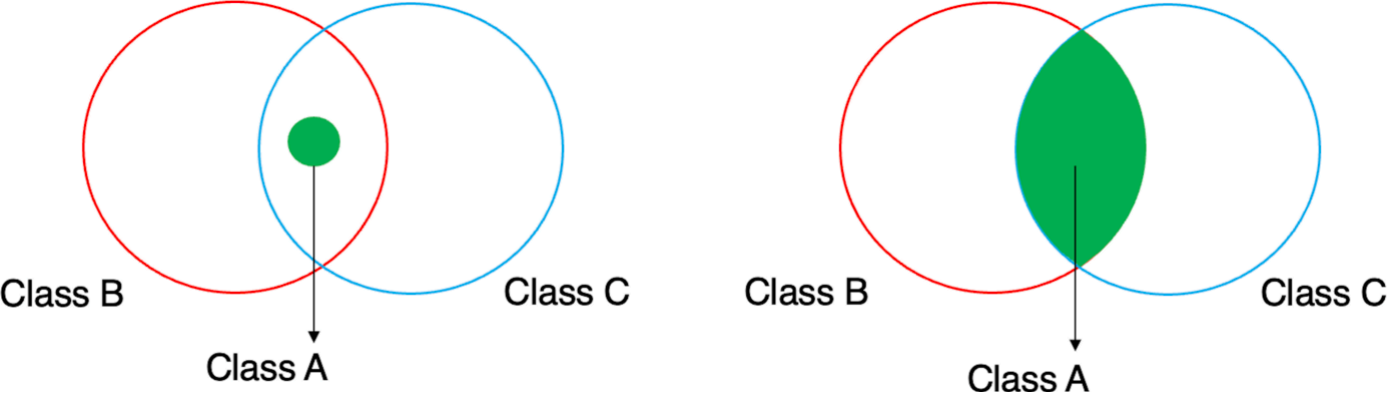
Illustration of the two different types of logical statements asserted in the new structure of PaNET. On the left, a weaker logical statement is shown, in which class *A* is only a subclass of the intersection of classes *B* and *C*. On the right, a stronger logical statement is made, equating *A* to be the entire intersection instead.

In the context of this paper, making stronger logical statements refers to replacing the subclass axioms in PaNET currently with equivalence axioms. The difference is illustrated in Fig. 3 ▸. Given an arbitrary class *A* with parent classes *B* and *C*, asserting the former to be a subclass of the latter two classes results in *A* being a subset of the intersection of *B* and *C*, *i.e.*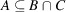. On the other hand, making *A* equivalent to *B* and *C* results in *A* being equal to the entire intersection of *B* and *C*, *i.e.A* ≡ *B* ∩ *C*. The latter is a stronger statement since it entails the former. Consequently, the stronger statement has more implications, enabling the reasoner to make more inferences.

However, care must be taken when deciding which subclass axioms to replace, as there are situations in which a stronger statement is logically inconsistent within the PaN science domain. To avoid this, the set of class subsumption axioms, on which PaNET is based, generated from these new equivalence axioms will be carefully validated against the current structure of PaNET before the changes are accepted. This is expanded on in Section 4.

Beyond the risk of introducing incorrect subclass relationships, a more subtle but significant issue remains: the possibility of embedding conceptual errors into the ontology. Using the same example from Fig. 3 ▸, class *A* is made equivalent to the intersection of classes *B* and *C*, based on the confident judgement that there cannot reasonably exist (now or in the future) any class that is a subclass of both *B* and *C* but is disjoint from *A*. Although these equivalence axioms reflect the current expert understanding, they must be made cautiously. A weaker statement, such as the subclass axiom, would not necessarily be incorrect and is the safer choice. However, such a statement reduces the reasoning capabilities of the new structure. For this reason, and given the reliability of expert consensus, the decision was made to accept this risk in pursuit of a more expressive and semantically robust ontology.

### 3.5. Redefining the technique classes

With these new concept classes and object properties, the technique classes have been redefined using stronger logical statements, shifting away from the explicit subclass relationship assertions in PaNET currently. How exactly they have been redefined depends on several factors. In general, there are three cases to consider:

(i) Core technique classes defined using existential quantifications. These classes have been made equivalent to the anonymous class expressions formed by existential property restrictions. These restrictions are constructed using object properties and concept classes, as explained in Section 3.3. For example, <refraction technique> is now equivalent to {(definedByProcess) **some** [refraction]}. Formally, the <refraction technique> class is now represented as follows [shown in Manchester syntax (Horridge & Patel-Schneider, 2012 ▸) for readability]:[Chem scheme1]
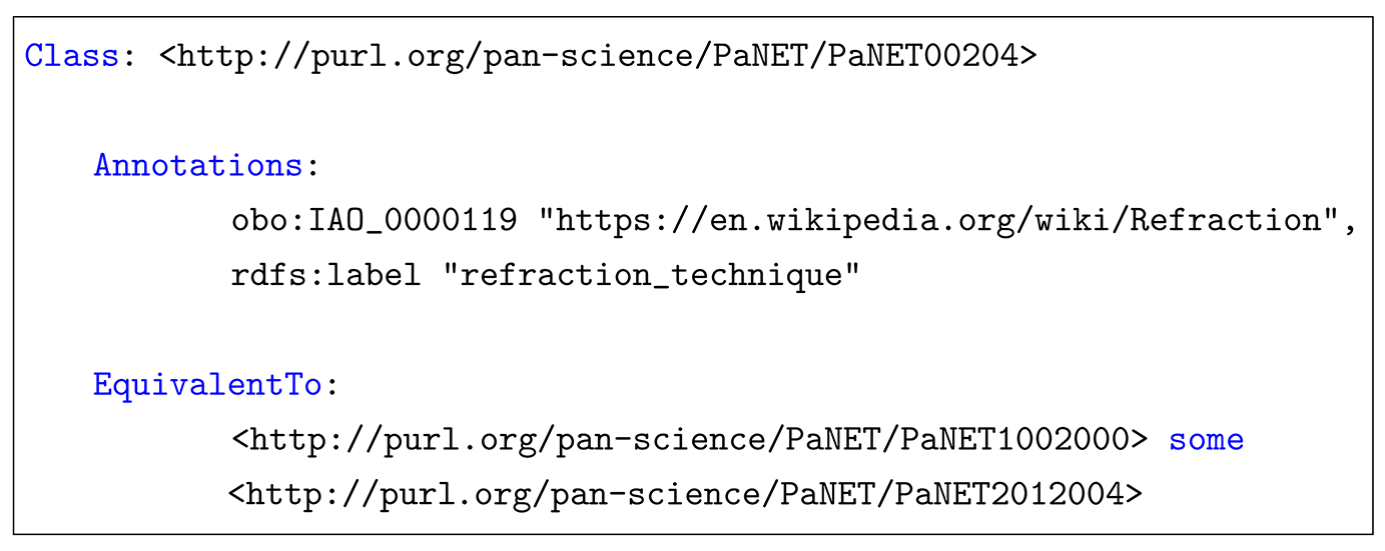


(ii) Non-core technique classes defined using equivalence axioms. These classes do not have a direct counterpart concept class and consequently cannot be defined in the same way as the core technique classes above. Instead, they have been asserted to be equivalent to the intersection of their current parent classes in PaNET. In other words, the subclass axioms currently in the ontology have been changed to logically stronger equivalence axioms. For example, <x-ray refraction imaging technique>, instead of being a subclass of, is now equivalent to the intersection of <refraction technique>, <x-ray probe technique> and <imaging technique>. These three classes are shown below as <PaNET00204>, <PaNET01012> and <PaNET01106>, respectively:[Chem scheme2]
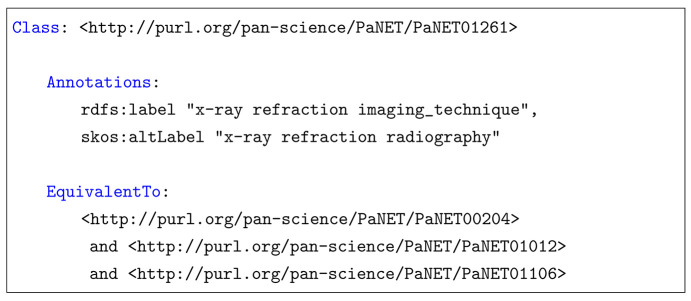


**Figure 4:**
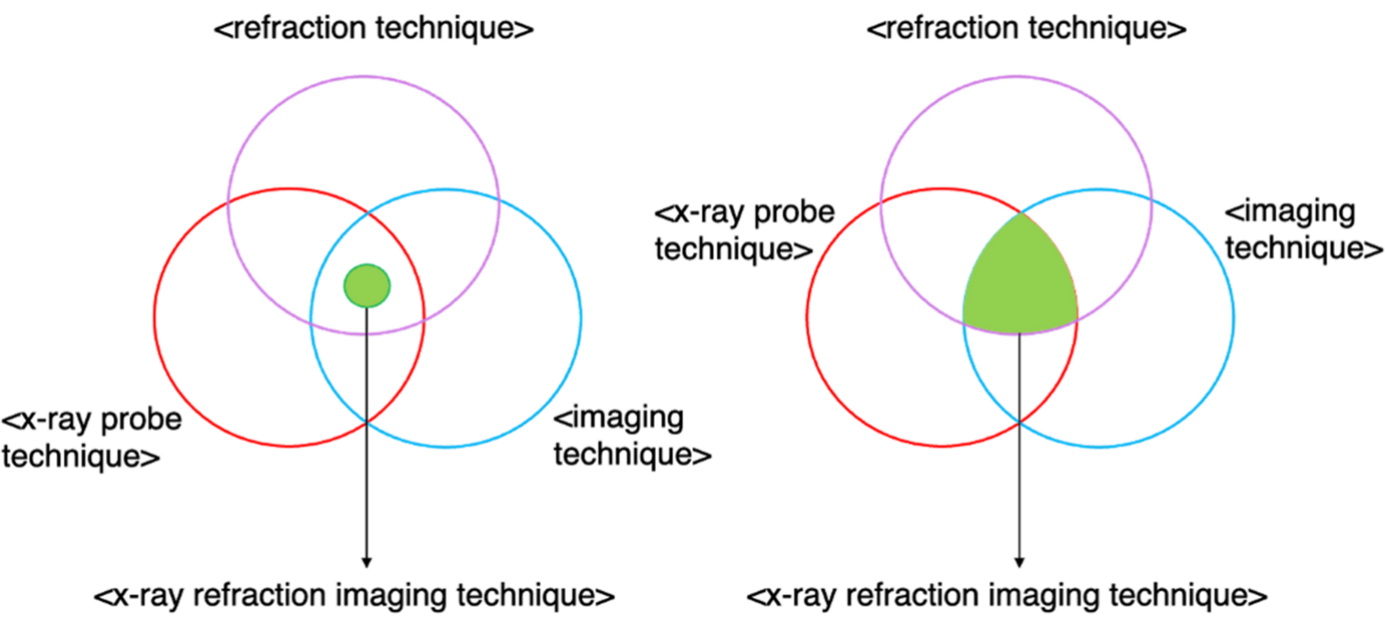
In PaNET, <x-ray refraction imaging technique> was asserted to be a subset of the intersection of three parent classes (left diagram). In the new structure of PaNET, it has instead been asserted to be equivalent to the entire intersection (right diagram).

This change in axioms is also illustrated in Fig. 4 ▸.

(iii) Non-core techniques defined using subclass axioms. The definitions for these classes remain the same as they currently are. This is because stronger logical statements would be inaccurate in their case. For example, two classes sharing the same set of parent classes would be inferred to be equivalent to each other if they were asserted to be equivalent to the intersection of the parent classes. This is the case for <versus photon linear polarization technique> and <versus photon circular polarization technique>, which share the same set of parent classes: <versus polarization technique> and <photon technique>. Another example would be classes that only have a single parent class; making them equivalent to their respective parent classes would be incorrect, as is the case with <natural linear dichroism technique> and its parent <linear dichroism technique>. In these cases, it is a sign that PaNET is still missing the building blocks that would allow differentiation between these techniques and thereby provide a stronger definition. Gradually, PaNET should evolve into an authoritative ontology capable of providing strong distinct definitions for all techniques.

Out of the 374 technique classes at the time of writing, 126 are core techniques defined using existential quantifications, 199 are non-core techniques defined using equivalence axioms and 49 remain defined using subclass axioms.

## 4. Validation

The validation of the proposed vision of PaNET is done by comparing its inferred subclass hierarchy with the manually constructed current hierarchy of PaNET. This is carried out using the *ROBOT* tool, specifically employing the diff command to compare two ontologies and output a text file listing the differences.

In this case, only differences in subclass relationships are relevant, as the goal is to make sure that the new structure of PaNET captures the entire pre-existing class hierarchy while also verifying any new relationships inferred by the reasoner that do not currently exist. Other differences, such as new classes and equivalence axioms, are expected and are not the focus of this comparison.

Through an iterative process, the proposed structure of PaNET is continually refined to ensure that it fully captures the current class hierarchy of PaNET, without missing any of the existing subclass relationships. The new inferred subclass relationships (see Section 4.2) are also scrutinized, with the more obviously correct relationships verified by the PaNET group and the more complex ones by domain experts. Incorrect relationships are then rectified by correcting the underlying logical axioms. This validation exercise has also flagged existing issues within PaNET (see Section 4.1).

**Figure 5:**
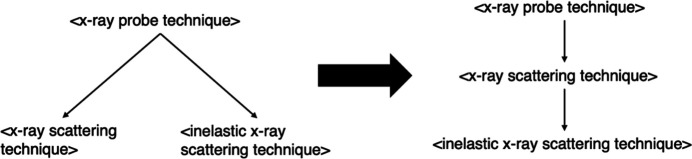
In PaNET currently, <x-ray scattering technique> and <inelastic x-ray scattering technique> are sibling classes that are both child classes of <x-ray probe technique>. In the new structure of PaNET, <x-ray scattering technique> becomes an intermediate class in between the other two. In essence, one direct subclass relationship currently in PaNET is no longer present in the proposed structure, corresponding to a new direct subclass relationship being added to the new structure.

The exact direct subclass relationships, or parent–child relationships, that are present currently do not need to exist in the new structure; rather, it is enough just for a subclass relationship to exist at all. For example, the *ROBOT* tool has picked up that <inelastic x-ray scattering technique> is currently a direct subclass of <x-ray probe technique> but that no such direct subclass relationship exists in the new structure. Instead, there is now an intermediate class <x-ray scattering technique> that exists in between them, such that <x-ray scattering technique> is the immediate ancestor of <inelastic X-ray scattering technique>, and <x-ray probe technique> is the immediate ancestor of <x-ray scattering technique>. This change is illustrated in Fig. 5 ▸. This also means that a new subclass relationship exists between <x-ray scattering technique> and <inelastic x-ray scattering technique>, which has also been observed by *ROBOT*. Hence, this is sufficient to verify that there remains a ‘path’ between <inelastic x-ray scattering technique> and <x-ray probe technique>, even if it includes intermediate classes. There is no need to check if the new path is correct as it will be verified when checking the list of new subclass relationships anyway, making the former redundant. This path-finding task is carried out using a graph traversal algorithm, specifically breadth-first search.

At the time of writing, the proposed new structure of PaNET resides in a fork of the main repository (see *Data availability* at the end of the paper for links). It will only be merged into the main repository once all of the issues outlined above have been resolved. Any errors that are overlooked and discovered later will be reported as issues in the main repository and addressed through the existing standard procedure. As the repository is publicly accessible, external contributors are also encouraged to submit issues.

### 4.1. Issues found in PaNET

A total of 17 issues in PaNET have been identified. For example, <diffuse scattering technique> should be a subclass of <scattering technique> rather than <elastic scattering technique>. Some have already been resolved in the new structure of PaNET to prevent logical inconsistencies. The rest have been submitted as issues on the GitHub repository for PaNET (Collins *et al.*, 2020 ▸). This ensures that these potential errors are carefully reviewed and addressed, facilitating better synchronization between the development of PaNET and the proposed structure.

### 4.2. New inferred subclass relationships

A total of 117 new subclass relationships have been inferred by the reasoner. Of these, 102 have been verified as correct, 6 require further expert validation, and the remaining 9 have been identified as likely incorrect.

This result reinforces the effectiveness of using automated reasoning over manual definitions to build the class hierarchy of PaNET. The 102 correct new relationships address both known and previously overlooked gaps in the completeness of the ontology. Although less obvious subclass relationships may still be missing, such omissions would likely stem from the evolving nature of science, reflected in changes to the underlying class definitions, rather than an unintended oversight of existing knowledge.

The 15 problematic relationships have been submitted as issues to the forked GitHub repository of the new structure of PaNET (see *Data availability* at the end of the paper), where they will be reviewed by the PaNET working group along with other domain experts. These relationships are most likely the result of applying overly strong logical statements (Section 3.4), in which case the relevant axioms will be reverted to a weaker form.

In Appendix *A*[App appa], an example of a correctly inferred subclass relationship is provided, along with an explanation of how it has been inferred by the reasoner.

## 5. Future of PaNET

The PaNET working group will continue to maintain and update the ontology. Additionally, the group will address the nearly 20 issues identified in this work before transitioning to the new structure of the ontology.

Work is also underway to migrate the existing PaNET GitHub repository to the Ontology Development Kit (ODK) standard (Matentzoglu *et al.*, 2022 ▸). The ODK is a set of tools, methodologies and workflows designed to streamline the management of ontology lifecycles. While primarily aimed at biomedical ontologies, it is still applicable to ontologies such as PaNET. This migration will streamline PaNET’s release process, standardize its metadata and align it with broader standardization efforts, ultimately enhancing its interoperability in accordance with the FAIR principles.

In addition, efforts are underway by various PaN facilities to build a PaN technique term selector service. Diamond Light Source has plans to integrate a PaNET term selector into Diamond DataGateway. At DESY, work has started on developing a web-browser-based technique selector. To support this development, an initial target app is being built (Nentwich, 2024 ▸) that allows users to view details of all DESY beamlines that offer the selected technique.

Other possible use cases are currently the subject of ongoing research. In the meantime, the casual user will continue to be exposed mainly to taxonomical aspects (subclasses and superclasses of techniques) along with annotation properties for human semantics. In this respect, there will be no change in the way PaNET is used, at least in the short term. While the new structure offers improved expressibility and enhanced semantics, the main motivation is to increase the completeness and correctness of PaNET as a taxonomy.

### 5.1. ESRFET ontology

The European Synchrotron Radiation Facility (ESRF) Experimental Techniques (ESRFET) ontology (De Nolf & Koumoutsos, 2024 ▸) is another effort in the same domain targeting only the photon-based techniques used in ESRF. The ESRFET ontology defines experimental techniques by focusing on the experimental process, including also the description of the sample used in the experiment. The goal of the ontology is to start by differentially defining techniques using their characteristics to build definitions. So, it always applies the OWL ‘equivalentTo’ axioms to define techniques and lets the reasoner calculate the subclassing tree. In that way, the erroneous and brittle manual subclassing is avoided. These characteristics typically include: (i) what kind of physical interaction the technique is based on (*e.g.* diffraction, fluorescence), (ii) what properties are being measured (*e.g.* energy, intensity), (iii) what kind of input is required (*e.g.*X-rays, electrons) and (iv) what kind of detector or measurement method is used (*e.g.* energy-dispersive detector).

By using this formal structure, the ontology allows researchers and machines to: (i) search for techniques based on specific properties, (ii) automatically classify or compare techniques and (iii) check whether a technique meets a set of criteria.

The ESRFET ontology uses PaNET and its classes by importing it and creating equivalent mappings between techniques. In that way, both ontologies will absorb each other’s embedded knowledge and behave as one when reasoning and SPARQL querying are applied. During this mapping process, semantic negotiations will be performed in order to resolve conflicting knowledge that the automated reasoning will reveal.

## 6. Summary

As the field of PaN science continues to evolve, maintaining and updating the PaNET ontology in its current form will become increasingly challenging. Use of the ontology has also exposed gaps in its completeness. The proposed vision of PaNET addresses these limitations by creating and incorporating the logical building blocks for a framework that enables automatic class hierarchy shaping, resulting in the addition of about a hundred subclass relationships that are currently missing from PaNET. Furthermore, this work has identified almost 20 further issues within PaNET.

These improvements position the PaNET ontology for future integration into PaN facilities, particularly to provide metadata richness in their data catalogue services, supporting the broader push towards FAIR data.

## Figures and Tables

**Figure 6:**
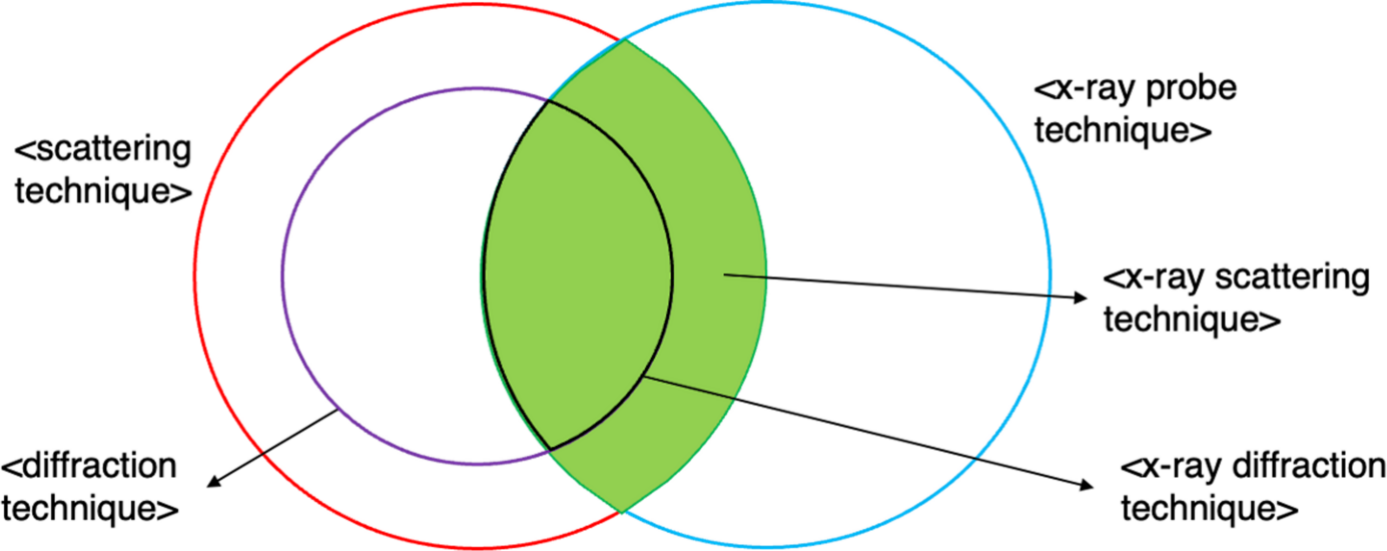
<x-ray scattering technique> is the intersection of <scattering technique> and <x-ray probe technique>, *i.e.* the entire shaded region. <x-ray diffraction technique> is the entire intersection of <diffraction technique> and <x-ray probe technique>, making it a subset of the shaded region, and thereby <x-ray scattering technique>.

## Data Availability

PaNET can be found on the following GitHub repository: https://github.com/ExPaNDS-eu/ExPaNDS-experimental-techniques-ontology. The proposed new structure of PaNET is currently on a forked GitHub repository: https://github.com/terencetan-c/ExPaNDS-experimental-techniques-ontology/tree/PaNET-Enhanced-Reasoning.

## Appendix A Explanation of an example inferred subclass relationship

The reasoner has inferred that <x-ray diffraction technique> is a subclass of <x-ray scattering technique>.

Axiom 1: [elastic scattering]  [scattering].

Axiom 2: [diffraction]  [elastic scattering].

Axiom 3: <scattering technique> ≡ {(definedByProcess) **some** [scattering]}.

Axiom 4: <elastic scattering technique> ≡ {(definedByProcess) **some** [elastic scattering]}.

Axiom 5: <diffraction technique> ≡ {(definedByProcess) **some** [diffraction]}.

Axiom 6: <x-ray scattering technique> ≡ <scattering technique> ∩ <x-ray probe technique>.

Axiom 7: <x-ray diffraction technique> ≡ <diffraction technique> ∩ <x-ray probe technique>.

From Axioms 1–5, Inference 1: <diffraction technique>  <scattering technique>.

From Axioms 6 and 7 and Inference 1, Inference 2: <x-ray diffraction technique>  <x-ray scattering technique>.

Inference 2 is also illustrated in Fig. 6 ▸.
